# Supplementary material for: The Proteome Profile of Olfactory Ecto-Mesenchymal Stem Cells-Derived from Patients with Familial Alzheimer’s Disease Reveals New Insights for AD Study
Source: Int J Mol Sci. 2023 Aug 9;24(16):12606. doi: 10.3390/ijms241612606 (PMC10454072; doi:10.3390/ijms241612606)
Supplement: Supplementary file 1 [file ijms-24-12606-s001.zip › supplementary figures.pdf]

Supplementary figures

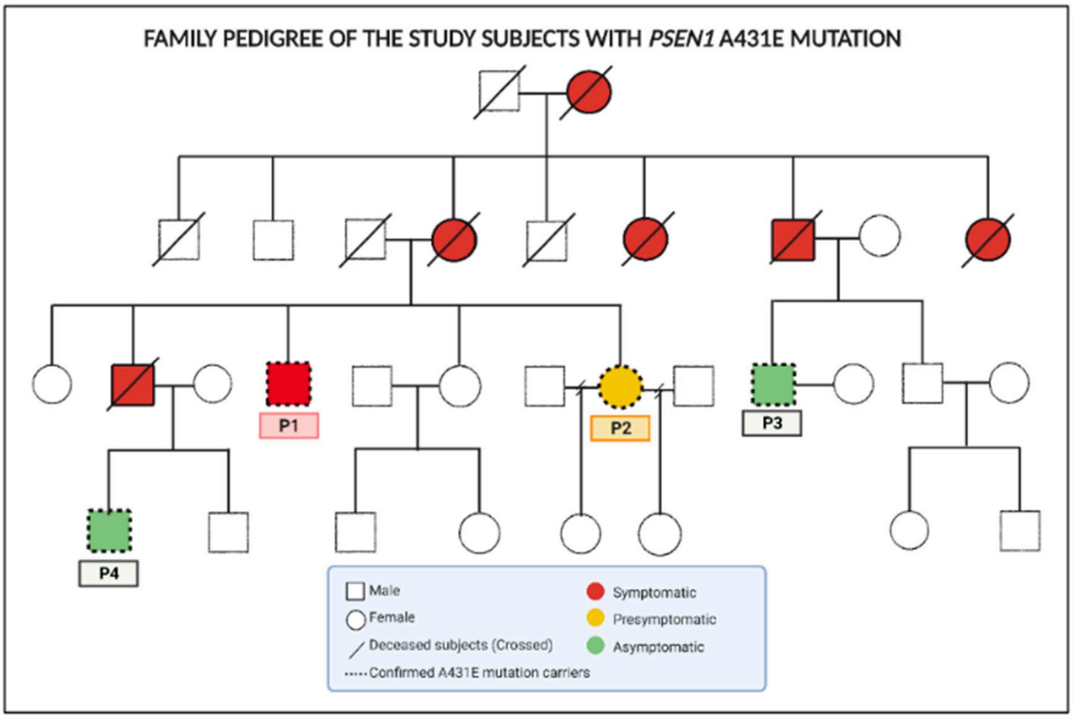

Figure S1. Family pedigree of the study subjects. Males are illustrated as squares and females as circles. Crossed figures depict deceased individuals. PSEN1 (A431E) mutation carriers genetically verified are shown with a dotted line [33].

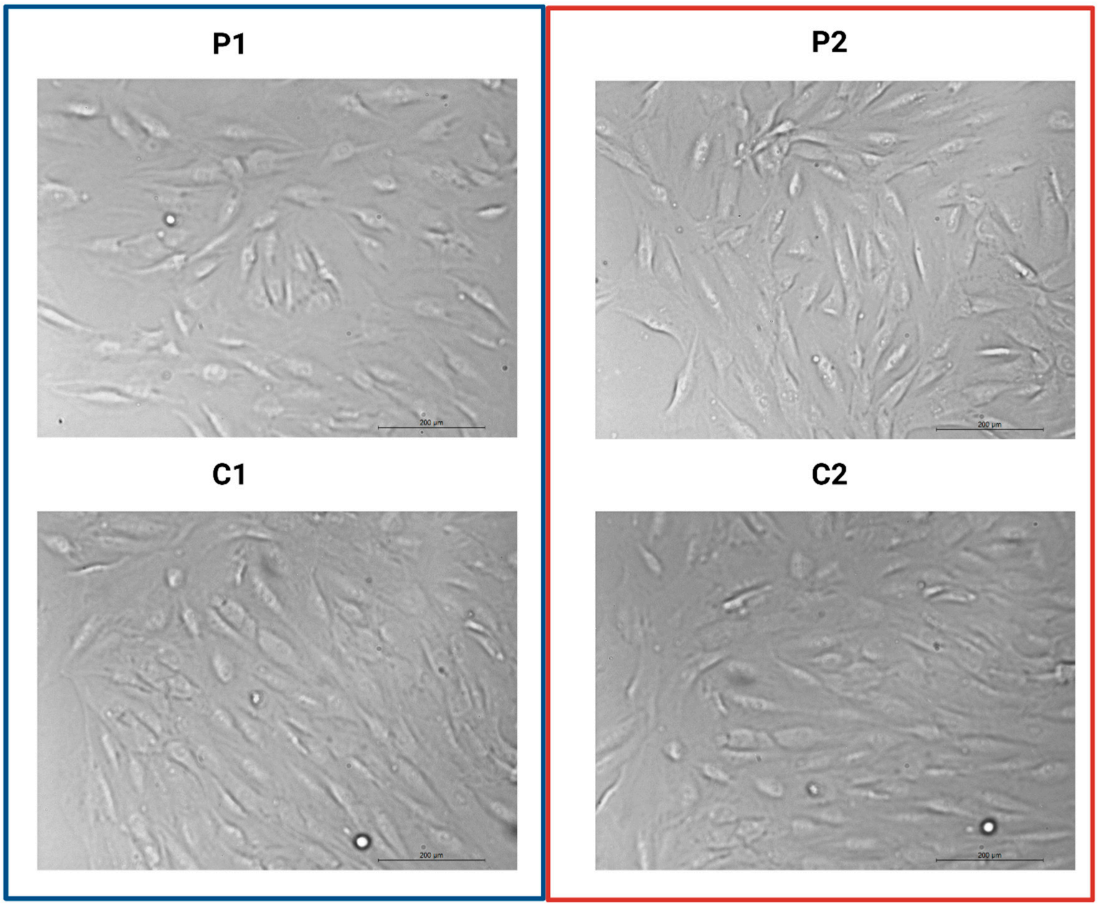

Figure S2. Cell morphology of study subjects. The left panel shows the cell morphology of P1 and C1, and the right panel shows the cell morphology of P2 and C2.

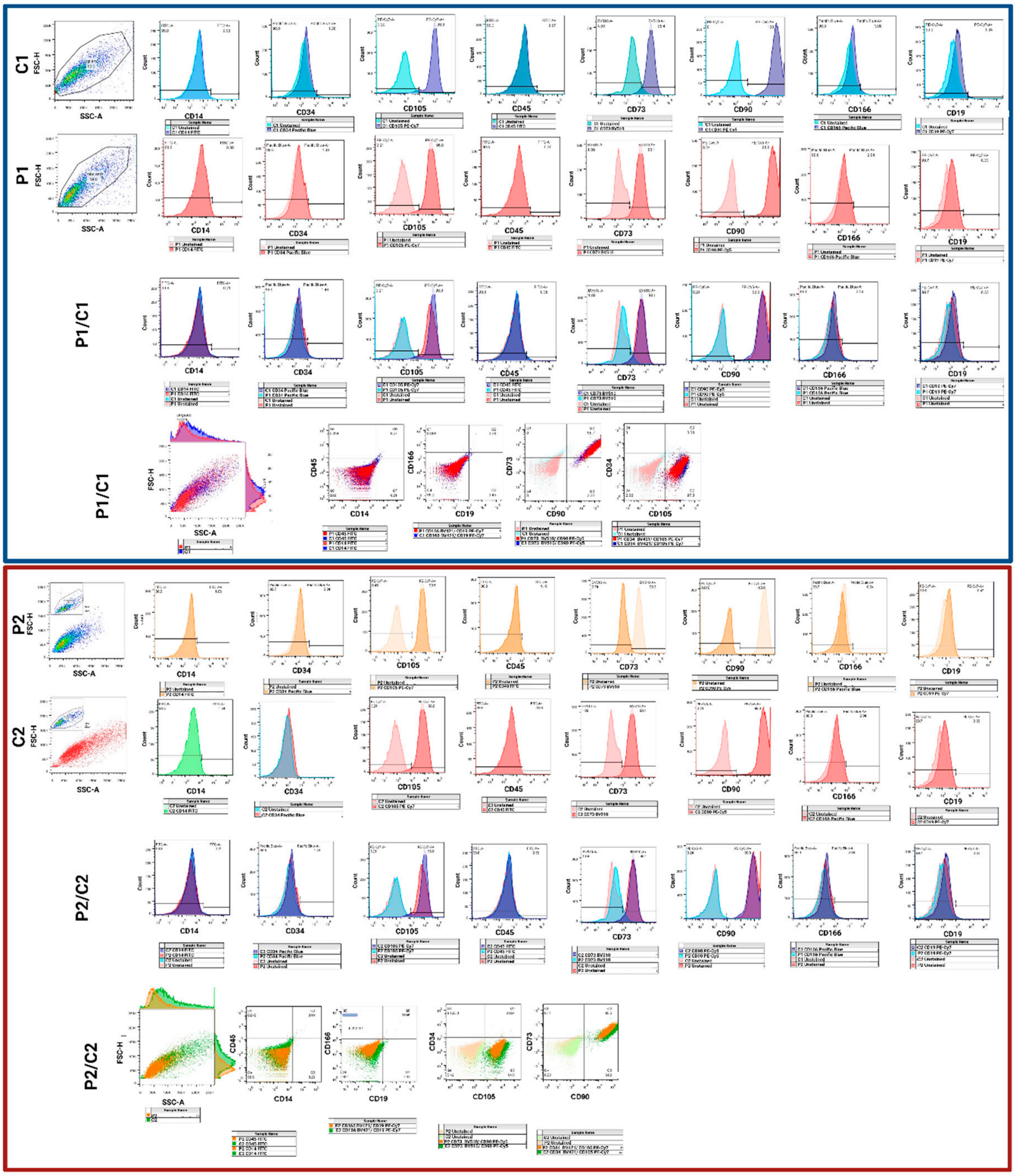

Figure S3. Histograms and dot plots of MSC markers. The blue square shows the histograms and dot plots of MSCs markers of P1 and C1 cells individually and merged. The red square shows histograms and dot plots of MSCs markers of P2 and C2 cells individually and merged. Cells were negative for hematological markers and differentiated cells CD34, CD45, CD14, CD19, and CD166 and positive for MSCs markers CD105, CD90, and CD73.
